# The Effect of Head Model Simplification on Beamformer Source Localization

**DOI:** 10.3389/fnins.2017.00625

**Published:** 2017-11-09

**Authors:** Frank Neugebauer, Gabriel Möddel, Stefan Rampp, Martin Burger, Carsten H. Wolters

**Affiliations:** ^1^Institute for Biomagnetism und Biosignalanalysis, University of Münster, Münster, Germany; ^2^Department of Sleep Medicine and Neuromuscular Disorders, Epilepsy Center Münster-Osnabrück, University of Münster, Münster, Germany; ^3^Department of Neurosurgery, University Hospital Erlangen, Erlangen, Germany; ^4^Institute for Computational and Applied Mathematics, University of Münster, Münster, Germany

**Keywords:** EEG, MEG, source analysis, beamformer, realistic volume conductor modeling, finite element method, epilepsy, kurtosis

## Abstract

Beamformers are a widely-used tool in brain analysis with magnetoencephalography (MEG) and electroencephalography (EEG). For the construction of the beamformer filters realistic head volume conductor modeling is necessary for accurately computing the EEG and MEG leadfields, i.e., for solving the EEG and MEG forward problem. In this work, we investigate the influence of including realistic head tissue compartments into a finite element method (FEM) model on the beamformer's localization ability. Specifically, we investigate the effect of including cerebrospinal fluid, gray matter, and white matter distinction, as well as segmenting the skull bone into compacta and spongiosa, and modeling white matter anisotropy. We simulate an interictal epileptic measurement with white sensor noise. Beamformer filters are constructed with unit gain, unit array gain, and unit noise gain constraint. Beamformer source positions are determined by evaluating power and excess sample kurtosis (g_2_) of the source-waveforms at all source space nodes. For both modalities, we see a strong effect of modeling the cerebrospinal fluid and white and gray matter. Depending on the source position, both effects can each be in the magnitude of centimeters, rendering their modeling necessary for successful localization. Precise skull modeling mainly effected the EEG up to a few millimeters, while both modalities could profit from modeling white matter anisotropy to a smaller extent of 5–10 mm. The unit noise gain or neural activity index beamformer behaves similarly to the array gain beamformer when noise strength is sufficiently high. Variance localization seems more robust against modeling errors than kurtosis.

## 1. Introduction

Magnetoencephalography (MEG) and electroencephalography (EEG) are non-invasive functional brain mapping tools with very high temporal resolution (Brette and Destexhe, 2012) and, in case of sufficiently realistic volume conductor modeling, also appropriate spatial resolution. They are thus useful to study highly dynamic neural activity. The solution of the EEG and MEG inverse problem is relying on the solution of the forward problem, i.e., the simulation of EEG and MEG for a given source in the brain. For the solution of the forward problem, the geometrical and electromagnetic features of the head need to be modeled, and every modeling also necessitates an appropriate simplification due to the high number of different head tissues and the inter- and intra-individual changes in conductivities (Haueisen et al., 1997).

The simplification used depends on the image data at hand, usually magnetic resonance imaging (MRI) and practical reasons as more detailed models require more time for segmentation and more sophisticated mathematical methods, and are therefore more labor-intensive and most often also computationally more expensive (Vorwerk et al., 2012).

A common approach segments the head into skin, bone, and brain, the so-called realistically-shaped three compartment head model (Kybic et al., 2005; Vorwerk et al., 2012; Stenroos and Nummenmaa, 2016). More realistic approaches further segment the brain into cerebrospinal fluid (CSF), gray matter, and white matter, as well as the bone into compacta and spongiosa (Ramon et al., 2004; Rice et al., 2013; Montes-Restrepo et al., 2014). Furthermore, white matter anisotropy can be modeled. In practice, diffusion tensor imaging (DTI) is used to gain anisotropy information (Tuch et al., 2001; Güllmar et al., 2010; Vorwerk et al., 2014).

Naturally, the question how much work is needed to get reliable results arises and is still debated today (Vorwerk et al., 2012). In this work, we want to investigate the effect of decreasing the number of modeled compartments in a realistic and geometrically correct head model on source analysis with beamforming methods.

Beamformers are inverse methods first used in radar and radio communication that have been adopted in brain research (Van Veen et al., 1997). For a given point a beamformer designs a spatial filter that passes signals originating at that position (and direction) and suppresses other signals and noise. These filters depend on the data covariance to optimize their properties.

Beamformers have the advantage that the number of sources does not have to be determined in advance (even though it should be lower than the number of sensors) as for dipole fitting methods nor do they smear focal sources like minimum norm solutions (Sekihara et al., 2005). Another advantage is their robustness to environmental noise, as each position is computed individually, noise parts of the measurement can be filtered without the need to explain the data in terms of goodness of fit. As such, the source space does not influence the calculation for a given position.

However, beamformers assume uncorrelated sources and show significant problems arising from source correlation. Beamformer are blind to fully correlated sources with their activity vanishing from their true position's reconstructed activity. If their combined signal is similar to a source's forward solution in the source space, it can be falsely reconstructed as a single source. This effect usually happens on spatially close sources (Van Veen et al., 1997; Sekihara et al., 2002).

As each filter reconstructs a source waveform, different further techniques can be used to find sources of interest or look for interesting features (Hillebrand et al., 2005). For localization the variance (or power) of the waveform is usually used, but excess kurtosis has been introduced to improve the localization of epileptic activity (Robinson et al., 2004; Kirsch et al., 2006). As epileptic spikes differ in their form from usual oscillatory brain activity, they form outliers in the data. Thus, kurtosis is often used to identify and localize them among brain noise that might have a greater power. Other works use brain connectivity measures to explore brain activity (Gross et al., 2001; Brookes et al., 2011; Hillebrand et al., 2012; Nissen et al., 2017).

In this work, we will focus on directional beamformer used for epileptic source localization. We will compare results for variance and kurtosis with different head models. Starting with a realistically shaped model with skin, skull, and brain, we will add CSF, white and gray matter distinction, and finally white matter anisotropy to get step wise more realistic models. Simulating two epileptic spikes in 60 different regions and both MEG and EEG, we use three kind of normalized linear constraint minimum variance beamformer to localize the activity on a cortical source space once with variance and once with kurtosis as localization criterion. We find that a simple normalization of the leadfield by its Frobenius norm is not enough to reliably localize activity in noisy data, while the commonly used neural activity index and the array gain beamformer by Sekihara and Nagarajan (2008) work similarly well for both the localization with variance and kurtosis.

We find that both modalities need to include CSF and white/gray matter distinction to localize with under 1 cm precision. The spongy bone mostly effects only EEG localization results, while both modalities profit from white matter anisotropy to a small extent of 5–10 mm. We find kurtosis to be less robust to modeling errors, making good head modeling especially necessary for epilepsy source reconstruction.

This work is structured as following: In section 2, we give a short overview of beamformer filter techniques, head model generation, finite element method modeling, and the details for our generation. In section 3, the results of our simulation are given. In section 4, we compare our results to previous works in forward modeling and inverse solutions. Furthermore, we address the limits of this simulation. We end with the short conclusion of our work.

## 2. Methods

Let **B** be the *N* × *T* measurement matrix of *N* sensors with *T* time samples. We assume that **B** has zero mean at every sensor. Here sensors can refer to both MEG magnetometers or gradiometers, EEG electrodes, or a weighted combination thereof. Then $\text{C}=\frac{1}{T}\overset{T}{\underset{t=1}{\sum }}\text{B}\left(t\right)\text{B}{\left(t\right)}^{T}$ is the *N* × *N* sample covariance matrix, where **B**(*t*) is the *N* × 1 measurement vector at time sample point *t*. For a position *Q* let **L**(*Q*) = (*L*^*x*^(*Q*), *L*^*y*^(*Q*), *L*^*z*^(*Q*)) the *N* × 3 forward solution (leadfield) for a dipole in each Cartesian (or any other orthogonal system) direction. Let *D*(*Q*) denote the true or estimated direction of *Q* and *L*(*Q*) = **L**(*Q*)*D*(*Q*) be the *N* × 1 leadfield of *Q* in direction *D*(*Q*). In the following equations, we will omit the position *Q* for better readability, as every position implies its own set of independent equations.

### 2.1. Beamformer filter design

The idea of a beamformer filter *W* is to suppress every type of signal except for one matching a given forward solution (Van Veen et al., 1997; Sekihara et al., 2005; Sekihara and Nagarajan, 2008). As this is mathematically impossible for arbitrary types of noise signals, beamformers adapt to the data to suppress only those sources of noise, that have been active during the measurement. Therefore, the output variance Var(*W*^*T*^**B**) = *W*^*T*^ **C** *W* is minimized subject to a constraint referring to the wanted signal. Here, *W* is a *N* × 1 vector corresponding to *N* × 1 forward solution. This is called a scalar beamformer, as *W*^*T*^**B**(*t*) is a scalar for every sample point *t*. We will focus only on scalar beamformers in this work, but note that vectorized versions can be constructed with only minor changes to the formulas (Van Veen et al., 1997; Huang et al., 2004; Sekihara and Nagarajan, 2008; Johnson et al., 2011).

The constraints for the wanted forward solution are

(1) $$W_{\text{ug}}^{T}L=1$$

(2) $$W_{\text{uag}}^{T}L=\left\|L\right\|$$

(3) $$W_{\text{ung}}^{T}L>0\text{ and }W_{\text{ung}}^{T}W_{\text{ung}}=1$$

for the unit gain, unit array gain, and unit noise gain constrained beamformer. These constraints lead to slightly different filters

(4) $$W_{\text{ug}}=\frac{{\text{C}}^{-1}L}{L^{T}{\text{C}}^{-1}L},$$

(5) $$W_{\text{uag}}=\left\|L\right\|\frac{{\text{C}}^{-1}L}{L^{T}{\text{C}}^{-1}L},$$

(6) $$W_{\text{ung}}=\frac{{\text{C}}^{-1}L}{\sqrt{L^{T}{\text{C}}^{-2}L}}.$$

Note that, given the same forward solution *L*,

(7) $$W_{\text{uag}}=\left\|L\right\|W_{\text{ug}},$$

(8) $$W_{\text{ung}}=\frac{W_{\text{ug}}}{\left\|W_{\text{ug}}\right\|}=\frac{W_{\text{ug}}}{\sqrt{W_{\text{ug}}^{T}W_{\text{ug}}}},$$

so Equations (5) and (6) are scaled versions of Equation (4). In this case, using Equation (6) is equivalent to the neural activity index used by Van Veen et al. (1997) and equivalent up to a constant factor to the pseudo-Z value, that is normalization by white noise of arbitrary strength, used by Vrba and Robinson (2001).

However, the source direction is generally unknown and needs to be determined by the data. The direction maximizing the output variance is used, which is generally different for each constraint. It can be derived analytically as a generalized eigenvalue problem (Sekihara et al., 2004; Sekihara and Nagarajan, 2008) as it forms a generalized Rayleigh quotient.

Thus, the direction can be computed without first computing a non-directed filter and directly used to obtain an optimal filter for the given data.

As the unit gain constraint is known to have a depth bias in presence of noise, a common way to implement the method is to normalize the three dimensional leadfield **L** by its Frobenius norm. Note that this leads to a similar form as the unit array gain, but does not enforce **L***D*_ug_ to have unit norm and does not effect the formula to calculate the direction. We have used this method of normalization only for the unit gain constrained beamformer.

### 2.2. Kurtosis

Excess kurtosis is a measure of tail heaviness of a distribution in comparison to the normal distribution with equal mean and variance (Westfall, 2014). For a sample vector *X*, its sample excess kurtosis is defined as the excess kurtosis of the sample distribution and given by

$${\text{g}}_{2}(X)=\frac{\frac{1}{T}\sum_{t=1}^{T}{\left(X_{t}-\bar{X}\right)}^{4}}{(\frac{1}{T}\sum_{t=1}^{T}{\left(X_{t}-\bar{X}\right)}^{2}{)}^{2}}-3,$$

where $\bar{X}$ is the mean of *X*. g_2_(*X*) can be used to find outliers in the data and it was shown that interictal epileptic spikes increase kurtosis in the data distribution (Kirsch et al., 2006; Livesey, 2007).

In this work, we use kurtosis instead of variance in the last step of the analysis, so the output of the beamformer is ${\text{g}}_{\text{2}}\left(W^{T}\text{B}\right)$ instead of Var(*W*^*T*^***B***). Note that the design of the filter and the calculation of the direction is not affected by this modification.

### 2.3. Model

#### 2.3.1. Model setup

To simulate a realistic head, a volume conductor with six compartments and white matter anisotropy was used.

A healthy 25-year old male subject gave written informed consent and all procedures have been approved by the ethics committee of the University of Erlangen, Faculty of Medicine on 10.05.2011 (Ref. No. 4453).

T1-weighted, T2-weighted, and diffusion-tensor (DT) MRI scans were acquired with a 3 T MRI scanner. MR images were resampled to a 1 mm isotropic resolution.

The skin, skull compacta, and skull spongiosa were segmented by applying a gray-value based active contour approach (Vese and Chan, 2002). Then the segmentation was manually corrected and foramen magnum and the two optic canals were correctly modeled as skull openings. The model was not cut off directly below the skull, but extended by skin up to the neck (Lanfer et al., 2012). The FreeSurfer-toolbox (https://surfer.nmr.mgh.harvard.edu) was then used to segment and extract the cortex surface of the white and gray matter interface.

In order to apply a constrained Delaunay tetrahedralization, all obtained surfaces were checked for intersections and those found were corrected by flattening the inner surface, ensuring a minimal distance between all surfaces. Using TetGen (http://www.tetgen.org) a mesh with 984,569 nodes and 6,107,561 tetrahedal elements was created. The tensor for white matter anisotropy was empirically computed using the approach of (Tuch et al., 2001; Rullmann et al., 2009; Ruthotto et al., 2012). Each element in the mesh was assigned the conductivity according to its barycenter.

For more details about model construction, we refer to Vorwerk et al. (2014).

#### 2.3.2. Head model

To investigate the influence of different head compartments on the accuracy of the beamformer inverse solution, we used models with different discrimination between these compartments. For every model, the geometrical structure is identical, that means that we do not model geometrical errors. As result, all models could be called realistically shaped. We constructed five different models with increasing accuracy, starting with the commonly used 3 compartment model, taking skin, skull, and brain matter into account. For the 4 compartment model the cerebrospinal fluid (CSF) surrounding the brain is separated from the brain matter. In the next step, white matter is distinguished from gray matter, further refining the brain compartment. For the 6 compartment model the skull compartment is divided into compact skull and spongiosa. For the last model used as a realistic reference, we include the white matter anisotropy in our model. The exact conductivities and an overview of the models is shown in Table 1.

**Table 1:** Overview of the compartment conductivities, the conductive features of the different head models (✓ is regarded, ✕ is disregarded, and d is regarded but further divided, A is anisotropic).

	**σ S/m**	**3CI**	**4CI**	**5CI**	**6CI**	**6CA**
Brain	0.33	✓	✓	d	d	d
Brain gray matter	0.33	✕	✕	✓	✓	✓
Brain white matter	0.14	✕	✕	✓	✓	✓A
CSF	1.79	✕	✓	✓	✓	✓
Skin	0.43	✓	✓	✓	✓	✓
Skull	0.01	✓	✓	✓	d	d
Skull compacta	0.0081	✕	✕	✕	✓	✓
Skull spongiosa	0.025	✕	✕	✕	✓	✓

#### 2.3.3. Finite element forward approach

We applied the finite element method (FEM) to solve the forward problem due to its ability to work with complicated geometries, such as skull holes, and tissue conductivity anisotropy without serious influence on computation speed and accuracy. In our study, we decided to use the Venant approach based on comparisons of the performance with other FE methods, such as the subtraction approach and the partial integration direct approach, and boundary element methods, as the symmetric BEM and the double-layer BEM (Vorwerk et al., 2012). However, the Venant approach relies on the assumption that a current dipole can be approximated by a set of monopoles near the dipole position with equal moment and overall zero charge. To fulfill this assumption, special care has to be taken when the source space is constructed. To find realistic positions we placed sources with a normal constraint on the gray/white matter and, if the closest vertex was not entirely part of the gray matter, moved them into the direction of the next valid node, until this node was the closest to the source. Thereby we ensured numerical accuracy, and that two sources would not be positioned on the same node (Vorwerk et al., 2014).

#### 2.3.4. Sensor setup

**Figure 1:**
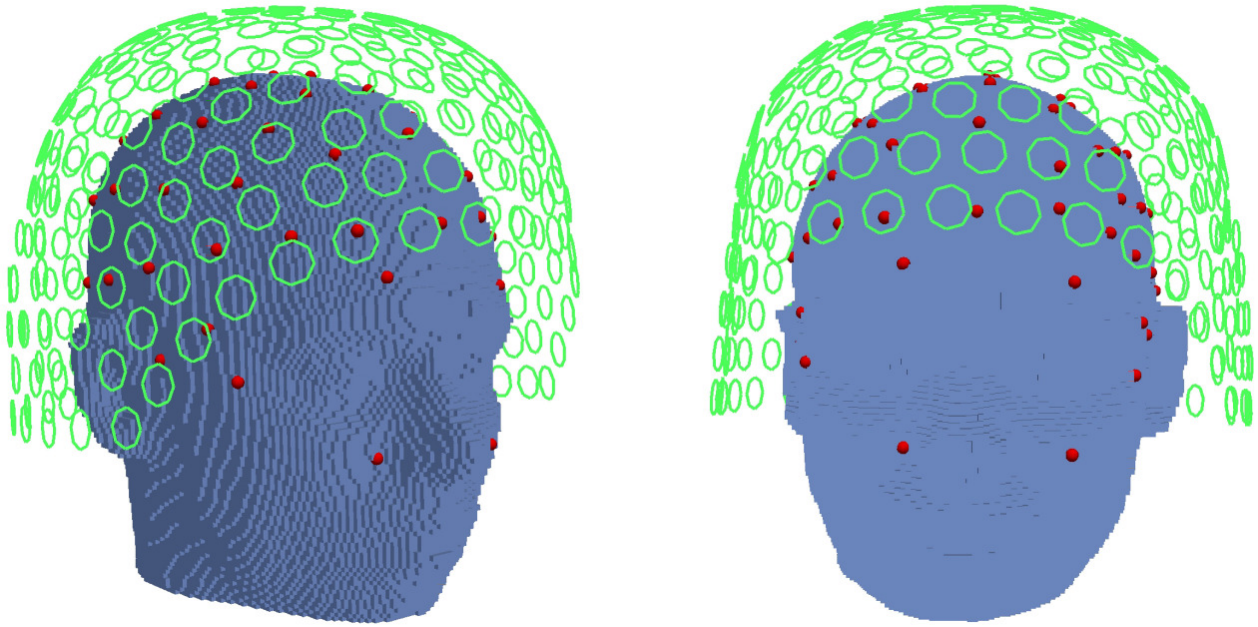
Position of the EEG electrodes (red spheres) and MEG gradiometers (green circle) in relation to the head. Only the first coil of each MEG gradiometer is shown.

In the following simulations, beamformer sensitivity with regard to volume conduction modeling was studied in EEG and MEG scenarios. We used a standard 10/10 EEG system with 80 channels and common average reference. For the MEG simulations, we used a whole head MEG system with 273 operational channels (CTF Omega 2005 MEG by MISL, http://www.vsmmedtech.com/hardware.html). The MEG system originally has 275 axial gradiometers and 29 reference sensors. The setup can be seen in Figure 1. EEG electrodes are depicted as red spheres, MEG gradiometers as green circles. Only the first coil of each gradiometer is shown, the second coils are placed in the outside direction of the circle normals.

#### 2.3.5. Source positions and source space

**Figure 2:**
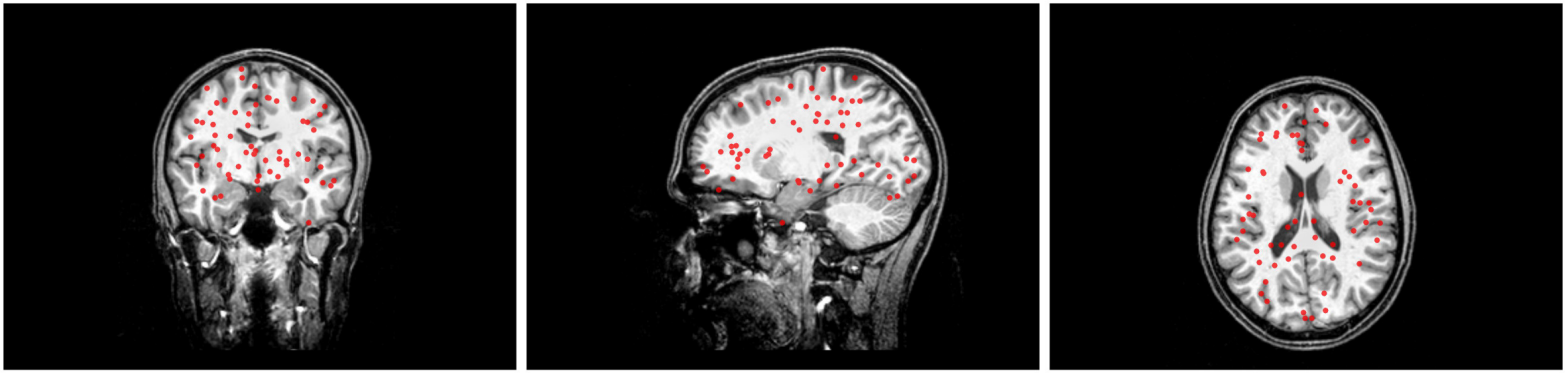
Position of the sources in the brain. Sources as dots, overlayed to a transparent MRI.

For the simulated reference sources, 60 points were placed on the cortex according to the constraint described in section 2.3.3 and MEG forward solutions were calculated. Their positions can be seen in Figure 2. Please note that the brain is set to be fully transparent, so all positions are seen regardless of their depth in the figure. Following Huang et al. (2007), source direction were then calculated as the direction of the eigenvectors corresponding to the smallest and biggest eigenvalue of the MEG forward solution matrix, corresponding to a quasi-radial and quasi-tangential direction. For the inverse source space used by the beamformers, the cortex was represented by 8,000 points, and forward solutions were calculated for every Cartesian direction, without any normal constraints. To prevent an inverse crime (Kaipio and Somersalo, 2006) while achieving a practical approximation, the beamformer source space had a minimum distance greater than 0.5 mm and smaller than 0.6 mm of the reference sources.

### 2.4. Simulation

**Figure 3:**
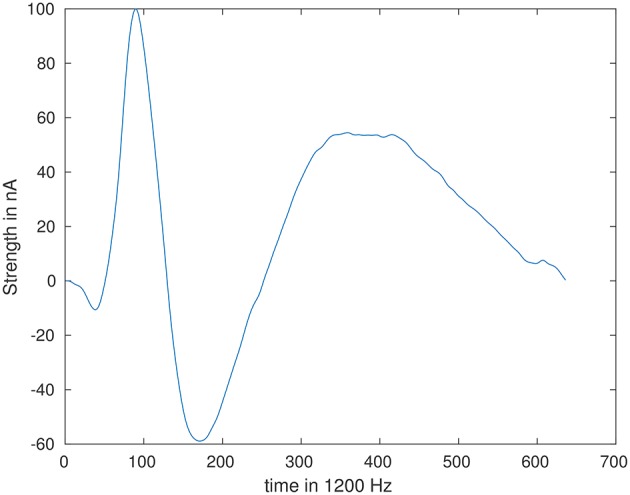
Simulated waveform of interictal spike.

To simulate a realistic epileptic spike source waveform, 15 marked interictal spikes of an epilepsy dataset were averaged and resampled at 1,200 Hz. The signal of electrode O1 was then used as a representative of a realistic source waveform. This waveform is 530 ms long (636 time samples) and is visualized in Figure 3. We simulated that the source with this source waveform and a maximum source amplitude of 100 nAm fired two times in 20 s (24,000 samples) long simulated measurement. All forward computations were done with the SimBio toolbox (SimBio Development Group, https://www.mrt.uni-jena.de/simbio), while the inverse analysis was implemented in Matlab (The MathWorks Inc. Version 2016a).

**Figure 4:**
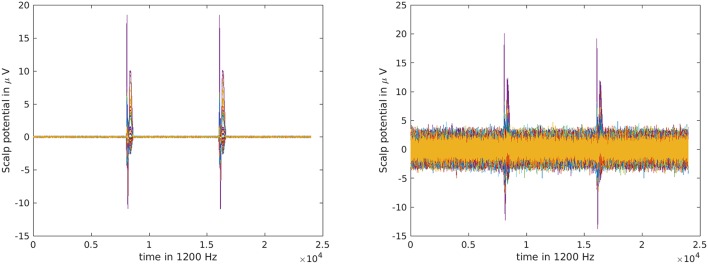
Simulated EEG measurement of one source point with low noise **(left)** and high noise **(right)** as butterfly plot.

For every sensor, random white noise was created with the Matlab randn function and scaled to have a variance of 10^−3^ and 1 μV^2^ for the EEG, and of 1 and 10^3^fT^2^ for the MEG simulation, creating a low and high noise scenario for both modalities. The same noise was used for all simulations, just varying the scale for the low and high noise scenario. In every simulation, only one source was active while the other stayed quiet. In the time between the spikes, no source was active. This led to optically equal measurement quality for MEG and EEG in tangential and radial direction, respectively. Two simulated EEG measurements are shown in Figure 4.

## 3. Results

The results are shown as box plots of the localization error. One box represents the extension of the error values for each model.

The median error is the value which separates the higher half of the errors from the lower half and is marked as a horizontal line. The lower quartile is the value that separates the lower quarter of the errors from the higher three quarters. Analogously, the upper quartile separates the higher quarter. The thick part of the box extends from the lower quartile to the upper quartile. The distance between the lower and upper quartile is called the interquartile range. The whiskers extend to the smallest/largest value with <1.5 times the interquartile range distance to the median or the minimum/maximum of the errors, whichever is larger/smaller. They are thus maximally 1.5 times as long as the box. Every value smaller/larger is considered as an outsider and marked with a plus sign. This is often called a Tukey boxplot (Frigge et al., 1989). The errors are capped at 40 mm to be able to distinguish small errors. If the median or outliers are above 40 mm, they are depicted on the dashed 40 mm line to remain visible.

Each box represents the errors of the simulation for one model, one noise strength, and one method of reconstruction. Each model is associated with a color to help to discriminate them.

As the beamformers work on a predefined source space, the localization error can never be zero. If we speak about a perfect or error-less reconstruction, it has to be understood as the best possible localization, that is with an error only due to the source space discretization between 0.5 and 0.6 mm. Values are rounded to mm for better readability.

### 3.1. Variance beamformer

#### 3.1.1. MEG

**Figure 5:**
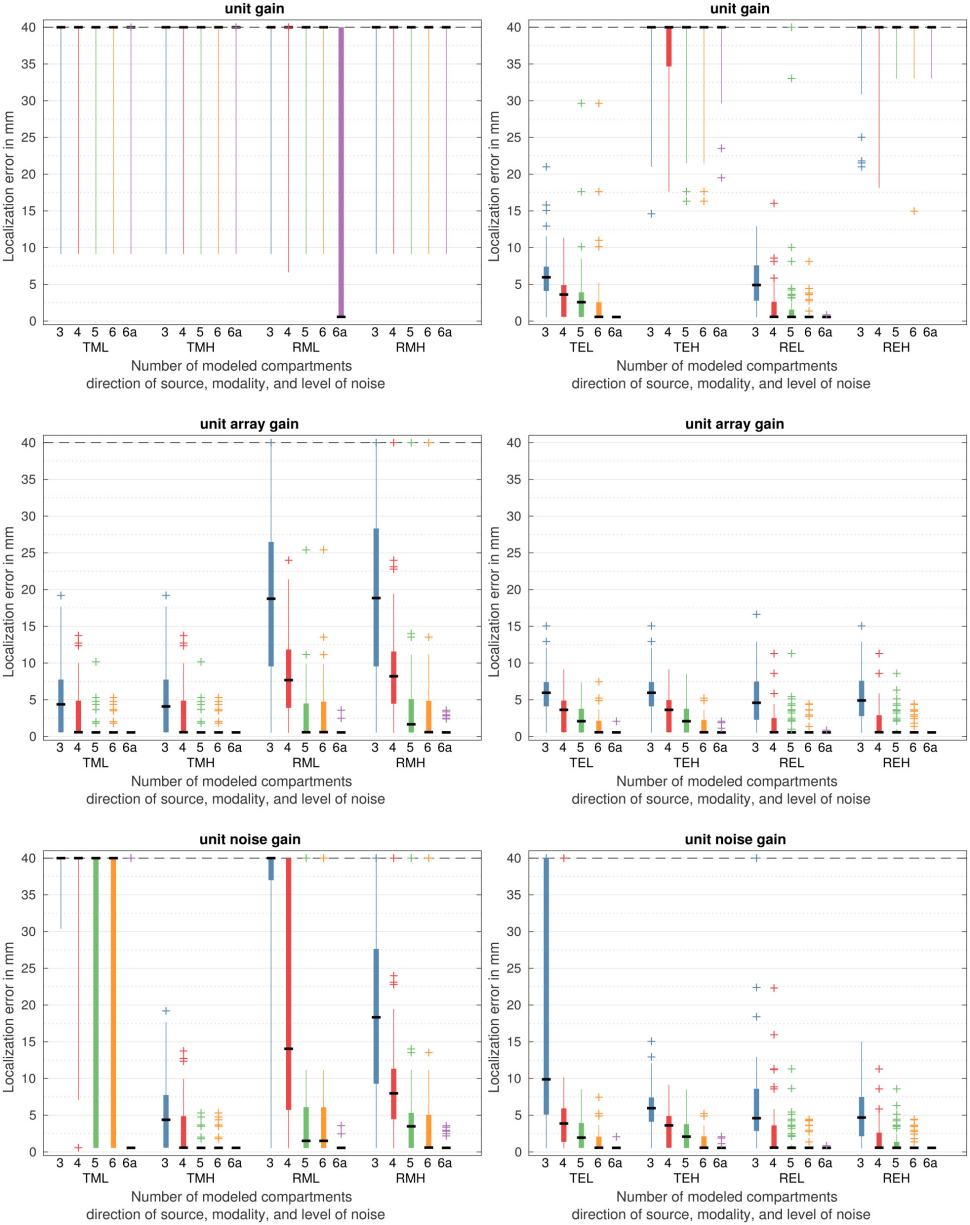
Localization errors based on the variance beamformers output for all head models. On the left side for MEG, right side for EEG. T for quasi-tangential, R for quasi-radial sources. L for low noise, H for high noise, M for MEG simulation, E for EEG simulation. Please refer to Table 1 for an overview of the models.

Figure 5 shows the results for all beamformer algorithms for MEG and EEG. MEG results are depicted on the left side.

The unit gain beamformer (top row) showed high errors in every simulation except for the reference model with median errors and even lower quartiles over 40 mm and no correctly reconstructed data. For the reference model the median was at the level of grid error, however, the upper quartile was still above 40 mm.

For the unit array gain beamformer (middle row) and the tangential source (middle row, TML and TMH) no difference between low and high noise was visible. For the 3 compartment model, the median error was at 4 mm and whiskers extend up to 18 mm. One outlier can be seen close to 19 mm. Including the CSF, that is extending from a 3 to a 4 compartment model, strongly reduced the error, yielding a perfect median error. Still, whiskers extended to 10 mm and outliers were up to 14 mm. Extending from 4 compartments to 5 compartments reduced the upper quartile to grid error, with only a few outliers in the 6 mm range and one at 10 mm. Modeling the spongiosa, that is going to 6 compartments, reduced the highest error from 10 to 6 mm, correcting one outlier. The reference model was able to localize every source correctly.

For radial sources (middle row, RML and RMH) the 3 compartment model showed a median error of 19 mm and an upper quartile of 27 mm, with whiskers stretching above 40 mm. Still, some sources were reconstructed with low error, even though the lower quartile was at 10 mm. Including CSF decreased the median error to 8 mm and the highest error to 25 mm, below the 3 compartment model upper quartile, in the lower noise scenario. However, for higher noise, there still was an outlier at 53 mm. Extending further to the 5 compartment model decreased the median to the grid error for the low noise and to 2 mm for the higher noise scenario. Whiskers still extended to 10 or 11 mm, and outliers existed at 25 and 55 mm, for low and high noise, respectively. Interestingly, these outliers were not the same sources for the 3, 4, and 5 compartment model, showing an increase in error for some sources with increasing accuracy. The 6 compartment model had an additional outlier in the low noise scenario, but decreased the median error in the high noise scenario to the grid error and had less outlier. It retained the high outlier at 55 mm from the 5 compartment model. The reference model could reconstruct all sources, except for two and three outliers for low and high noise, respectively. These outliers were below 5 mm error.

The unit noise gain beamformer (bottom row) performed nearly identical to the unit array gain beamformer in the high noise scenario (bottom row, TMH and RMH), except for a slightly higher median error for radial sources using the 5 compartment model.

For low noise and tangential sources (bottom row, TML), the median error and lower quartile decreased with increasing model accuracy, but median errors were above 40 mm for all models except the reference. The 5 and 6 compartment model had a lower quartile close to the grid error, but still showed a high spread of errors for nearly all sources. The reference had six outliers between 97 and 123 mm, reconstructing every other source without errors.

For radial sources and low noise (bottom row, RML), the same trend was visible, but with reduced errors for higher model accuracy. The 3 compartment model had a median and lower quartile above 35 mm, reconstructing only some sources without error. Including CSF yielded a lower median error of 14 mm, however, the lower quartile was still above 5 mm, still yielding errors for most sources, and the upper quartile was above 40 mm. The 5 and 6 compartment model did not differ for most sources, yielding a low median error of 2 mm and an upper quartile at 6 mm. Still, there were outliers far above 40 mm, up to 115 mm. The reference model had two outliers below 4 mm error and yielded no errors otherwise. Its performance was the same as for the unit array gain beamformer.

#### 3.1.2. EEG

The results for the EEG simulation can be seen in Figure 5 on the right side.

For tangential sources and low noise the unit gain beamformer (to row, TEL) showed a trend of decreasing error with increasing model accuracy. The 3 compartment model had a median error of 6 mm, a lower quartile of 4 mm, and an upper quartile of 7 mm. Outliers were up to 21 mm. Using the 4 compartment model reduced the median error to 4 mm and the upper quartile to 5 mm. No error above 11 mm occurred, giving the lowest maximum error of all models except for the reference. The 5 compartment model had a median of 3 mm, but yielded outliers at 18 and 30 mm, giving worse results than the 4 compartment model for some sources. With the 6 compartment model the median was reduced to the grid error, but outliers as for the 5 compartment model persisted. The reference 6 compartment anisotropic model was errorless.

In the high noise scenario (top row, TEH), however, no model could reconstruct any source without error. Even for the reference model the lowest error was an outlier at 20 mm and the median was 61 mm.

For radial sources the same overall behavior could be observed. For low noise the median (top row, REL) was perfect for all models except the 3 compartment model. The upper quartile decreased from 8 mm for the 3 compartment model to 3 mm for the 4 compartment model, and to 2 mm for the 5 compartment model. The 5 compartment model, however, had outliers up to 40 mm that did not exist for the 3 and 4 compartment models. The 6 compartment model still had outliers up to 8 mm, while the reference model had one outlier at 1 mm, reconstructing every other source without error.

For high noise (top row, REH) no model achieved a better localization than 15 mm, showing similar errors as for tangential sources.

The unit array gain beamformer showed the best results in all scenarios, steadily decreasing errors with increasing modeling accuracy.

For tangential sources (middle row, TEL and TEH) the 3 compartment model showed no errors larger than 15 mm with a median of 6 mm. The median was reduced to 4 and 2 mm for the 4 and 5 compartment model, respectively. For the 6 compartment and reference model, it was further reduced to the grid error. No model showed outliers with a greater error than the upper quartile of the next simpler model. The reference was not perfect, giving outliers of 2 mm.

For radial sources (middle row, REL and REH) the same trend could be observed with slightly better results for all models. The median was at the grid error for every model except the 3 compartment model. The upper quartile was at the grid error for the 5, 6, and the reference model. Many outliers up to 12 and 4 mm still persisted for the 5 and 6 compartment model.

The high and low noise scenario only differed in some outliers by a few millimeters.

The unit noise gain beamformer showed a stronger influence of noise than the array gain beamformer, but nearly identical behavior at higher noise.

For low noise and tangential sources (bottom row, TEL), the median error of the 3 compartment model was at 10 mm, but the upper quartile was at 54 mm and the maximum error at 133 mm. For high noise the error (bottom row, TEH) was at 6 mm and the maximum at 15 mm, giving the algorithm a better performance at more noisy data. Higher model accuracy improved performance and yielded almost the same results for both noise levels with median errors of 4 mm, 2 mm, and the grid error for the 4, 5, and both 6 and the 6a reference model, respectively. However, the 4 compartment model had outliers up to 104 mm for low noise. While the 6 compartment model had less outliers for higher noise, the reference model had more outliers in the high noise scenario, with errors of 2 mm for both noise levels.

For radial sources (bottom row, REL and REH) similar, but generally better, results could be observed. The 3 compartment model had outliers up to 100 mm with a median of 5 mm. For high noise the model had the same median, but a lower upper quartile and no outliers. All other models had a median at the grid error with an upper quartile at the grid error for the 6 compartment and the reference model. Outliers persisted for all models except for the reference at high noise. These outliers, however, were relatively low, with errors below 12 mm for high noise and below 23 mm for low noise and more than 3 compartments.

### 3.2. Kurtosis beamformer

#### 3.2.1. MEG

**Figure 6:**
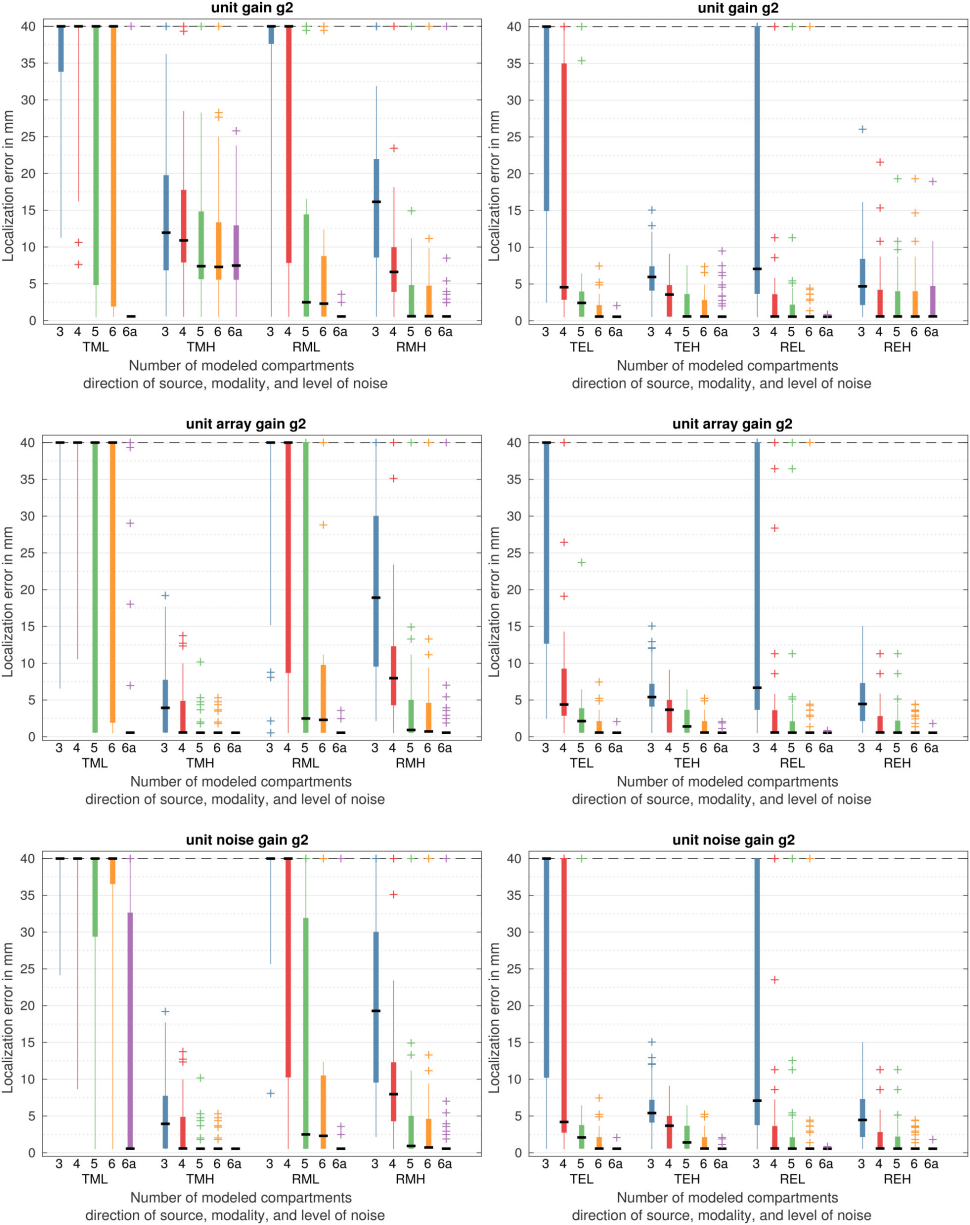
Localization errors based on the kurtosis beamformers output for all head models. On the left side for MEG, right side for EEG. T for quasi-tangential, R for quasi-radial sources, L for low noise, H for high noise, M for MEG simulation, E for EEG simulation. Please refer to Table 1 for an overview of the models.

Figure 6 shows the localization error of the beamformers using kurtosis instead of variance as the output. MEG simulation results are depicted on the left side.

The unit gain g_2_ beamformer showed great errors for tangential sources. In the low noise scenario (top row, TML) the median was above 40 mm for all models but the reference. While the lower quartile was lower for the 3 compartment model than for the 4 compartment model, both could not reconstruct any source without error, giving 11 and 8 mm as the best result. The 5 compartment model had a lower quartile of 5 mm, but the median was still at 65 mm. The 6 compartment model had slightly better results with a lower quartile of 2 mm and a median of 65 mm. While the reference had a median of the grid error, given perfect results except for some outliers.

For high noise (top row, TMH) all models performed very similarly with overall significantly spurious localizations, but lower errors than for low noise (top row, TML). The 3 compartment model had a median of 12 mm and an upper quartile of 20 mm. Adding CSF could improve the median to the grid error and the upper quartile to 11 and 18 mm, but outliers persisted up to 87 mm. The 5 compartment model had a median of 7 mm and an upper quartile of 15 mm, while the 6 compartment model improved the upper quartile to 13 mm. Both had outliers above 40 mm of 108 and 67 mm. The reference yielded comparable errors with a median of 8 and a lower quartile of 5 mm, but did not show outliers above 26 mm. For this model, the performance was far worse than for low noise.

For radial sources and low noise (top row, RML), the 3 compartment model yielded great errors with a median of 62 mm and a lower quartile of 38 mm. While the best performance was at the grid errors, only few sources achieved good localization. The 4 compartment model had a lower quartile of 8 mm, reducing the errors for many sources. Still, the median was at 46 mm. The 5 compartment model reduced the median to 3 mm and had an upper quartile of 14, giving a substantial reduction in errors. The 6 compartment model had a lower quartile of 9 mm, slightly reducing the errors further. Still, both had outliers of 127 and 121 mm. The reference had two outliers at 2 and 3 mm and yielded no errors otherwise.

For higher noise (top row, RMH), the same trend could be observed, but with overall better performance. The 3 compartment model had a median of 16 mm, which was reduced to 7 mm for the 4 compartment model and to the grid error for the 5, 6, and reference model. On the other hand, all models had outliers of 69 mm and the 4 compartment model even above, of 104 mm.

The unit array gain g_2_ beamformer performed similarly to the unit gain beamformer g_2_ for tangential sources and low noise (middle row, TML). With medians of 79, 77, 60, 66 mm for the models in increasing accuracy, performance was slightly worse than for the unit gain. The reference yielded 13 outliers up to 126 mm, giving no errors otherwise.

For high noise (middle row, TMH), however, the median errors were at 4 mm for the 3 and at the grid errors for the higher accuracy models. The maximum error decreased from 14 to 10 for the step from 4 to 5 compartments and to 5 for the step to the 6 compartment model. The reference yielded no errors.

For radial sources the performance was slightly worse than the unit gain for both high and low noise.

For low noise (middle row, RML), the 3 compartment model had a median of 67 mm, and only one outlier was reconstructed with no error. The 4 compartment model had a median of 44 and a lower quartile of 7 mm reconstructing most sources with errors above 10 mm. The median of the 5 compartment model was at 3 mm, while the upper quartile was still at 41 mm. The 6 compartment model, however, had a median of 2 mm and reduced the upper quartile to 10 mm. Still outliers of 128 mm persisted. The reference had outliers at 3 and 4 mm and yielded no errors otherwise.

For high noise (middle row, RMH), the median of the 3 compartment model was at 19 mm with outliers up to 96 mm. The 4 compartment model had a median of 8 mm and outliers of up to 112 mm. The 5 and 6 compartment models had a (rounded) median of 1 mm, with upper quartiles of 5 mm. Both had outliers of 111 mm, yielding great errors for some sources. The reference had a median at the grid errors, small outliers up to 7 mm, and one far outlier of 111 mm.

For unit noise gain g_2_ beamformer and for tangential sources and low noise (bottom row, TML), the medians of the localization were 81, 77, 69, 68 mm, and minimal for the models in order of increasing accuracy. With an upper quartile of 33 mm, the reference model's performance was the worst of all three tested beamformer approaches.

For high noise (bottom row, TMH), the performance was the same as for the unit array gain g_2_ beamformer (middle row, TMH).

For radial sources and low noise (bottom row, RML) we could observe a similar behavior as for the unit array gain g_2_ beamformer. The medians were 83, 53, 3, 2 mm, and minimal, again in order of increasing accuracy. Thus, they were greater for the 3 and 4 compartment model and the same for 5 and 6 compartment model for the unit noise gain g_2_ beamformer in comparison to the unit array gain g_2_ beamformer. However, the upper quartile of the 5 compartment model is at 32 mm, lower than for the unit array gain g_2_ Ḟor the 6 compartment model, the upper quartile is 1 mm higher, giving no large differences between the approaches. The reference had an additional outlier of 108 mm to the unit array gain.

For high noise the unit noise gain g_2_ beamformer (bottom row, RMH) performed exactly as the unit array gain g_2_ beamformer (middle row, RMH).

#### 3.2.2. EEG

Figure 6 shows the localization error of the beamformers using kurtosis instead of variance as the output. EEG simulation results are depicted on the right side. For the EEG, all methods performed with very similar median errors for most models.

The unit gain g_2_ beamformer showed better results for the EEG than for the MEG. For tangential sources and low noise (top row, TEL), the 3 compartment model had a median of 50 mm and a lower quartile of 15 mm. The 4 compartment model had a median error of 5 mm, performing already much better than the 3 compartment model. Still, the upper quartile at 35 mm was rather high. The 5 compartment model had a median of 2 mm, performing much better, except for outliers of 35, 55, and 124 mm. The 6 compartment model had no errors above 8 mm with a median at the grid error. The reference model had an outlier of 2 mm, yielding no errors otherwise.

With high noise (top row, TEH), errors were reduced when compared to low noise (TEL), but the same trend in accuracy could be observed. The 3 compartment model had a median of 6 mm and a maximum error of 15 mm, achieving a good overall localization. The 4 compartment model had a median of 4 mm and a maximum of 9 mm. The median of the 5 compartment error was at the grid error, with an upper quartile of 4 mm and a maximum of 8 mm. Adding the spongiosa to the model reduced the upper quartile to 3 mm and the maximum to 7 mm, while further adding white matter anisotropy, resulting in the reference model, reduced the upper quartile to the grid error. However, eleven outliers with errors up to 10 mm could not be perfectly reconstructed.

For radial sources and low noise (top row, REL), the 3 compartment model had a median error of 7 mm and an upper quartile of 42 mm, with outliers up to 136 mm. The 4 compartment model performed a lot better with a minimal median and upper quartile of 4 mm. Still, outliers up to 116 mm persisted. The 5 compartment model had an upper quartile of 2 mm, and the 6 compartment model of the grid error. Still, both models yielded outliers of 116 mm. Aside of an outlier of 1 mm, the reference mode yielded no error.

For high noise (top row, REH), the 3 compartment model had a median of 5 mm, while all other models had a median of the grid error. The upper quartiles of the 4, 5, and 6 compartment models were at 4 mm. The reference model had an upper quartile of 5 mm, giving a slightly worse result, while outliers were similar with a maximum of 20 mm across the 5 and 6 compartment models, and the reference model.

The unit array gain g_2_ beamformer showed a similar performance.

For tangential sources and low noise (middle row, TEL), the median errors were at 44, 4, and 2 mm for the 3, 4, and 5 compartment model, respectively. With a minimum error of 2 mm, the 3 compartment model was never errorfree. The 6 compartment model had a median at the grid error and maximum error of 8 mm, giving a good localization. The reference had an outlier of 2 mm and was errorless otherwise.

For high noise (middle row, TEH), the medians were at 5, 4, and 1 mm for the 3, 4, 5, compartment model. The 6 compartment model performed nearly identical, with a minimal median error and outliers at 6 mm. While the reference gained two additional outliers below 2 mm, it still performed errorless otherwise.

For radial sources and low noise (middle row, REL), the performance was nearly identical for the unit gain g_2_ and unit array gain g_2_ beamformers (top row, REL).

The 3 compartment model had a median error of 7 mm and an upper quartile of 44 mm, with outliers up to 125 mm. The 4 compartment model showed a much better performance with a minimal median and upper quartile of 4 mm. Still, outliers up to 121 mm persisted. The 5 compartment model had an upper quartile of 2 mm, and the 6 compartment model of the grid error. Still, both models yielded outliers of 122 mm. Aside of an outlier of 1 mm, the reference mode yielded no error.

For high noise (middle row, REH), however, the unit array gain g_2_ beamformer performed better than the unit gain g_2_ beamformer. The median for the 3 compartment model was at 5 mm, while all other models achieved minimal median error. While the 4 and 5 compartment model only differed in a slightly decreased upper quartile, the 6 compartment had a perfect upper quartile and only outliers below 5 mm. The reference model had one outlier at 2 mm, and was perfect otherwise.

The unit noise gain g_2_ beamformer yielded higher errors for tangential sources and low noise (bottom row, TEL). The 3 compartment model had a median error of 48 mm and an upper quartile of 82 mm. The median error of the 4 compartment model was at 4 mm, but the upper quartile was still at 46 mm. With a median of 2 mm and an upper quartile of 4 mm, the 5 compartment model had a much better result. Still, it yielded an outlier of 52 mm. The 6 compartment model corrected this outlier, yielding a perfect median error and a maximum error of 8 mm. The reference was errorless except for an outlier of 2 mm.

For high noise, the unit noise gain g_2_ beamformer (bottom row, TEH) performed exactly like the unit array gain g_2_ beamformer (middle row, TEH) described above.

For radial sources (bottom row, REL and REL) the unit noise gain g_2_ beamformer performed nearly like the unit array gain g_2_ beamformer for low noise and exactly like it for high noise.

The 3 compartment model had a median error of 7 mm and an upper quartile of 80 mm, with outliers up to 140 mm. The 4 compartment model a a much better performance with a minimal median and upper quartile of 4 mm. Still, outliers up to 112 mm persisted. The 5 compartment model had an upper quartile of 2 mm, and the 6 compartment model of the grid error. Still, both models yielded outliers of 106 mm. Beside on outlier of 1 mm, the reference mode yielded no error.

For high noise (bottom row, REH), the the unit noise gain g_2_ beamformer performed exactly like the unit array gain g_2_ beamformer described above (middle row REH).

## 4. Discussion

In this work, we investigated the influence of head model accuracy on the localization ability of different beamformer techniques. We used maximum variance beamformers with the unit gain, unit array gain, and unit noise gain (or neural activity index) constraint. Furthermore, we used both kurtosis and variance as output and localization criterion. For the influence of the head volume conductor model accuracy, we used a cascade of geometrically correct volume conductor with increasing number of compartments, starting with the commonly used 3 compartment model and ending with a 6 compartment model with white matter anisotropy.

The three beamformer methods we applied were all normalized variants on the linear constrained minimum variance (LCMV) (Van Veen et al., 1997) beamformer to deal with noise in the data. The applied normalization with the Frobenius norm was not sufficient to overcome the noise bias of the non-normalized LCMV, as the unit gain beamformer yielded large errors in the high noise scenario. The unit noise gain beamformer on the other side seems to need noisy data to work in practice, as it yielded high errors for low noise.

This can either be because of signal leakage, that is the spread or migration of the signal reconstruction to another position, or by filtering the data too sharp, as the signal can be interpreted as noise for every forward solution. The good localization using the reference model strongly points to the last interpretation, and the same effect was found by Hillebrand and Barnes (2003).

Another explanation would be errors due to numerical instability. As the covariance matrix is considered invertible only in the case of sufficiently strong noise, rank deficiencies in the matrix can cause errors in the inversion. While we cannot exclude inversion errors to be responsible for the localization errors, the covariance matrix and thus its inverse did not change across the algorithms. Thus, their sensitivity to covariance inversion can be considered as part of their robustness to noise. To address the rank deficiency of the covariance matrix, regularization techniques can be used. The simplest form, called diagonal loading, adds a scaled identity matrix to the covariance matrix before inversion. As perfect white noise yields a scaled identity matrix as its covariance matrix, diagonal loading can be considered as adding perfectly white sensor noise to the data. As we added simulated sensor noise, the difference between a clear signal with diagonal loading and our simulated noise scenarios is only due to numerical inaccuracy for the variance beamformers. Thus, diagonal loading can be expected to improve the performance at low noise to the level of high noise or above. However, this adds the question of the optimal diagonal loading factor to the analysis, which cannot be answered without further knowledge.

As our high noise scenario is a more realistic scenario for non-averaged data, we do not expect rank deficiency to have a large influence in praxis. If more spikes should be averaged, however, our work provides an argument against using beamforming on averaged data. Instead, event-related beamforming (Cheyne et al., 2007; Mohamed et al., 2013) offers a method to avoid matrix regularization while using the improved signal-to-noise ratio due to averaging.

In many practical applications, kurtosis and variance beamformers do not directly compete in localization ability. However, the epileptic brain activity differs in its shape from oscillatory brain rhythms and is therefore detectable by kurtosis. While variance beamforming is sensitive to the power of sources and can thus be widely used, it has the risk of favoring a stronger, non-epileptic source over an epileptic one.

Kurtosis, while shown to be more sensitive to modeling errors, is more specialized for sharp epileptic spikes and should thus be more robust to oscillatory brain noise. In practice, both methods should be used keeping their advantages and disadvantages in mind.

For the kurtosis (g_2_) beamformers, the difference in normalization is disregarded, as kurtosis is invariant to scalar multiplication. Interestingly, that gives the array gain kurtosis a worse performance at low noise compared to high noise, similar to the unit noise gain beamformers. The chosen direction, though, is based on variance output power and is thus still different. The relatively bad performance of the unit gain g_2_ beamformer points to a not optimally chosen direction in comparison to the other methods, which work almost identically. Furthermore, Prendergast et al. (2013) have shown that the direction for optimal variance is not the same as for optimal kurtosis, giving another criterion for the optimal direction and way of improvement. Another method is to include geometrical knowledge about the shape of the cortex and the medically plausible direction, but Hillebrand and Barnes (2003) have shown that small errors in cortex modeling give worse results than using no constraint.

As epilepsy surgery is not done based only on source analysis, their is no clear criterion for the goodness of the localization. In most studies, the quality of source localization is evaluated after surgery. It is then called successful, if the patient is seizure free and the reconstructed position is inside the resected zone, see for example (Brodbeck et al., 2011). As the resected zone will consist of a few cm^3^, errors in the millimeter range might be neglected. Depending on the exact procedure and the shape of the cortex, errors between 1 and 2 cm might still be acceptable, but bear more risk for a false hypothesis and problems with the further evaluation. In risky regions close to the eloquent cortex, centimeters can decide whether a surgery is possible or not. Furthermore, pointing at the true position opens the possibility to retrospectively reevaluate the MRI in search for lesions (Aydin et al., 2017).

The differences between the radial and tangential sources in our simulation are entirely due to the sensitivity of the modalities to their orientations, since their positions, amplitudes, and corresponding sensor noise are exactly equal. As expected, the MEG showed a higher sensitivity to tangential sources, while the effect of orientation was very low for the EEG. Source depth is critical for both MEG and EEG signal strength, as the SNR decreases with distance to the sensors. Especially for the MEG, however, the difference between radial and tangential sources is stronger for superficial sources (Hunold et al., 2016), so source depth might often be the decisive point for localization ability, when a realistic model is used.

In terms of model accuracy, the commonly used 3 compartment model gave reasonably results with less than 2 cm error for the MEG and the unit array gain beamformer, when the source was tangential. For more radial sources the model was not accurate enough to give usable localizations for about half of the sources. For the EEG, the 3 comaprtment head model gave reasonably good results for both radial and tangential sources.

For the kurtosis method, however, the 3 compartment model yielded larger localization errors for both modalities, which shows the need for better modeling when using g_2_ beamformers for spike localization.

Including the CSF in the model showed the greatest effect on source localization for both MEG and EEG and both variance and kurtosis output for the unit array gain and unit noise gain. Without including it, perfect localization was not possible and localization with <5 mm error rather uncommon. This is consistent with the errors of the forward solution described in Vorwerk et al. (2014). While our CSF effects are based on pure computer simulation studies, Rice et al. (2013) have proven its important effect on EEG in an experimental study. They have shown the effect of CSF shift due to head position, finding significant effects of small changes in CSF thickness, thus implying the importance of geometrically and compartment wise correct modeling. Bijsterbosch et al. (2013) found similar effects of head orientation due to subarachnoid CSF distribution. In another experimental study, Bangera et al. (2010) found the inclusion of CSF and white matter anisotropy to be important for an accurate description of the electric field inside the skull measured by intracranial EEG.

Furthermore, the CSF conductivity is well known and does not change significantly between subjects (Baumann et al., 1997), giving another practical reason to include it in the head model.

The effect of gray and white matter distinction was similarly strong as that of CSF modeling in both MEG and EEG. Especially for weaker sources such as radial sources in the MEG, no reliable localization was possible. This is again consistent with the differences in forward modeling found by Vorwerk et al. (2014) and Haueisen et al. (2000), who showed a significant influence of the conductivity of the surrounding area of a source. Van Uitert et al. (2004) showed a strong influence of gray and white matter conductivity especially on the magnetic field. If white matter is not distinct from gray matter, its conductivity is overestimated, thus leading to mistakes in forward and inverse solutions. While these authors used a dipole model, the same effect is seen in our beamformer approaches.

In our simulations, distinguishing compact from spongy bone was less important than the other effects discussed above. For variance based beamformer localization, the effect on the MEG was negligible except for the high noise scenario and radial sources, which showed an improvement of the median, if not for the maximum error. For the EEG, an improvement of about 5 mm could be seen for most sources. Interestingly, the effect is stronger for the kurtosis based localization. The same effect strength was observed for the forward solution by Vorwerk et al. (2014). Like in their study, we also used the optimized conductivity value for the homogenized skull that represents the best fit to the realistic spongiosa/compacta scenario (Dannhauer et al., 2011). Steinsträter et al. (2010) investigated the influence of skull modeling on EEG source localization with the unit noise gain beamformer, finding accurate geometry and skull conductivity to be necessary for accurate localization. However, they modeled the skull as a single anisotropic compartment, while we modeled it here as a three compartment (spongy bone enclosed by inner and outer compacta) tissue, which is a better model for the skull (Dannhauer et al., 2011; Montes-Restrepo et al., 2014). Still, we can see very similar results for both methods, as could be expected. Like (Dannhauer et al., 2011; Montes-Restrepo et al., 2014) we advise to include the spongiosa in the head model for EEG, if the resolution of the underlying MRI is sufficient. Since the additional work is rather minor, yet often manual, including it for the MEG as well might not be necessary.

Here, we used the true skull conductivity for the forward model, while in practice the individual skull conductivity is generally unknown. To avoid errors due to a wrong statistical assumption and especially for using combined MEG and EEG, using the MEG as a baseline for skull conductivity calibration is therefore recommended (Fuchs et al., 1998; Huang et al., 2007; Aydin et al., 2014).

White matter anisotropy showed a small, but sometimes substantial effect on localization ability for both modalities. Depending on source orientation, the effect varied from effecting some outlier sources to a substantial amount, especially for radial sources and MEG. While the strong influence for the radial sources is certainly partially due to the worse signal strength compared to the tangential orientation, the effect was also present in the work of Anwander et al. (2002). It was explained by the authors that tissue anisotropy only affects the secondary (conducting) currents and that the ratio of the secondary to the whole magnetic flux increases with the ratio of the radial dipole orientation component. They found mean localization errors of 5.1 and 8.8 mm for 43 radial and 46 tangential sources, respectively. While we give the median of the errors, as they are more robust, the tendency is consistent with our findings. Güllmar et al. (2010) performed a detailed investigation of white matter anisotropy, finding similar effects, albeit overall weaker. They explained this by the position of the sources, which highly influence the effect of the anisotropy. In an experimental study, Bangera et al. (2010) found the white matter anisotropy to be necessary to accurately model the electrical field measured by intracranial EEG. However, modeling the white matter anisotropy is clearly less important than including CSF and distinguishing gray and white matter compartments. For many applications, it might be neglected without strongly influencing the localization accuracy. If, however, the localization is not consistent with other knowledge about the probable origin of the source, this additional modeling step should be taken if a DTI with sufficient resolution is available, before MEG or EEG findings are rejected.

As this is a simulation study with only one head model, the depicted effects might vary from subject to subject. The overall agreement with other studies, however, seems to validate our methods. As we used the 6 compartment anisotropic model as reference for a realistic head, we neglected the influence of other tissues present in the brain, e.g., dura mater (Ramon et al., 2014). The 6 compartment model should thus not be thought of as fully realistic or complete.

As signal strength is different for each source due to the different sensitivities on source depth and orientation, and the difficulty to find a standard sensor noise strength for the EEG, the comparison of MEG and EEG localization ability is not entirely fair. This work should therefore not be considered as competition between the modalities.

Here we tested only for localization errors. However, as recent work has shown (Güllmar et al., 2010; Vorwerk et al., 2014), volume conduction effects are especially also influencing source orientation and strength, which have to be further investigated in future work with regard to the effect on beamformer reconstruction.

Further investigations should also be performed to compare beamformer methods with other inverse approaches such as current density reconstruction (CDR) methods. However, as investigated in Lucka et al. (2012), the classical minimum norm estimation (MNE) and also weighted MNE (wMNE) CDR methods suffer from depth localization deficits, while beamformers were shown to achieve high spatial accuracy (Sekihara et al., 2005). There, the spatial resolution for standardized sLORETA, which is known not to suffer from depth localization errors in single source scenarios (Pascual-Marqui, 2002; Sekihara et al., 2005; Lucka et al., 2012), was compared with the minimum-variance beamformer, and it was shown that the minimum-variance filter attains much higher resolution than sLORETA.

As beamformers use data segments instead of a short time or even one time point, like dipole fitting methods do, the simulation of source is more complicated. As we only did a one source study with rather low normally distributed noise, the effects in practice might be more pronounced due to spatially distinguished noise and multiple active sources in one trial. In that case, the choice of trial length will be much more important than in our simulation. Furthermore, we only simulated one run of white noise, giving a rough idea about the modeling effects rather than a statistic. However, as we discussed above, our results are consistent with other studies.

In future studies, it will still be necessary to test the influence of more realistic noise on the method, either using a patch model for a simulation of brain noise, or by directly using real data. This can be done with a phantom (e.g., Leahy et al., 1998), animal model (e.g., Lau et al., 2016), or in epilepsy patient investigations where the sources are well-known (e.g., Brodbeck et al., 2011). The later would also test the generality of our results, using many different subjects to construct the models. Additionally, the effect of combining MEG and EEG should be researched, as it showed better performance than the single modalities (Aydin et al., 2014; Hunold et al., 2016).

## 5. Conclusion

We showed that a realistic head modeling has strong effects on both EEG and MEG source localization with beamforming methods of more than 2 cm for many sources. For a realistic level of noise, both the array gain beamformer and the unit noise gain (neural activity index) beamformer worked similarly well. We strongly advise against using a unit gain beamformer, as its chosen direction is far from optimal. As the source strength and position is usually unknown, we advise to model at least the CSF and gray/white matter distinction, because many sources cannot be localized correctly without them. While the additional computational work is not neglectable, the risk of an otherwise possibly erroneous analysis should outweigh this additional effort. Modeling the skull compacta and spongiosa as different compartments is less important, but should still be considered for EEG analysis, as the additional amount of work is rather small. For the MEG this can be disregarded in most cases.

White matter anisotropy showed smaller effect on the localization ability, but still improved localization ability and might have a bigger effect on source orientation and strength reconstructions. As it increases the complexity of the model significantly, it might, however, be admissible to disregard white matter anisotropy as long as the results stay consistent with other knowledge about the source.

## Author contributions

FN and CW conceived the study. FN implemented the algorithms and simulation setup. FN performed the analysis, produced the images, interpreted the results, and wrote the paper. All authors took part in the scientific discussion at multiple stages of the study and provided feedback from the mathematical (CW, MB) and clinical (GM, SR) perspective. All authors reviewed the manuscript and approved it for publication.

### Conflict of interest statement

The authors declare that the research was conducted in the absence of any commercial or financial relationships that could be construed as a potential conflict of interest.
